# Electro-acupuncture for treatment of knee pain from osteoarthritis and the possible endocrinology changes: a study protocol for a randomized controlled trial

**DOI:** 10.1186/s13063-015-0766-2

**Published:** 2015-06-03

**Authors:** Javier Mata, Sandra Cabrera, Pilar Sanchís, Pedro Valentí, Patricia Hernández, Regina Fortuny, Serafin Lirola, Jose Luis Aguilar

**Affiliations:** Anaesthesiology and Clinic Pain Department, Son Llàtzer Hospital, Ctra. de Manacor kilómetro 4, 07198 Palma de Mallorca, Spain; Catalan Institute of Oncology, Germans Trias I Pujol Hospital, Ctra. Del Canyet s/n., 08916 Badalona, Spain; Research Unit, Son Llàtzer Hospital, Ctra. de Manacor kilómetro 4, 07198 Palma de Mallorca, Spain; Clinical and Haematology Analysis Laboratory, Son Llàtzer Hospital, Ctra. de Manacor kilómetro 4, 07198 Palma de Mallorca, Spain; Traumatology Department, Son Llàtzer Hospital, Ctra. de Manacor kilómetro 4, 07198 Palma de Mallorca, Spain

**Keywords:** Electro-acupuncture, Knee osteoarthritis, Chronic pain, Osteoarthritis, Beta-endorphin, Non-puncturing needle, Cortisol

## Abstract

**Background:**

Osteoarthritis of the knee is a major cause of disability among adults. Electro-acupuncture is considered a potentially useful treatment for osteoarthritis. The purpose of this study is to assess the efficacy of electro-acupuncture on pain control, pain perception, plasma cortisol and beta-endorphin levels, patient-perceived quality of life, and pain medication use in patients with chronic knee pain.

**Methods/design:**

This study is a placebo-controlled, randomized, double-blind, parallel design trial. One hundred sixty out-patients who are more than 50 years old and who have osteoarthritis of the knee will be recruited from the island of Mallorca, Spain. Each participant will be randomly placed into one of two groups: (sham) electro-acupuncture non-insertion technique and real electro-acupuncture.

Acupuncture treatments will be the Traditional Chinese Medicine type. The patients will be evaluated after a period of 1 month (with two weekly sessions), 3 months (with one monthly session), 6 months (with one session every 45 days), and 1 year later with follow-up sessions at the end of the study (with one session every 2 months).

The primary outcomes will be based on the observed changes from the baseline of the visual analogue scale (VAS) and the Western Ontario and McMaster Universities Osteoarthritis Index (WOMAC) for pain measured at 12 weeks after the end of treatment.

Also to be included in the study are the possible changes in the secondary efficacy variables from baseline as assessed by the Short Form 36 version 2 health survey (patient-perceived quality of life), patient plasma cortisol and beta-endorphin levels at the different treatment stages, the Goldberg Anxiety and Depression Scale, pain medication use, functional capacity and stiffness (WOMAC subscales), and a VAS. These variables will be assessed at 1 month, 3 months, 6 months, and 1 year after study commencement.

**Discussion:**

The findings from this study will help to determine whether electro-acupuncture is effective for chronic knee pain management in older people and whether electro-acupuncture can deliver results for the improvement of pain relief, stiffness, and disability. The study will therefore be a major step toward understanding the roles of the hypothalamic-pituitary-adrenal axis and the endogenous opioid system in the effectiveness of electro-acupuncture for chronic pain.

**Trial registration:**

ClinicalTrials.gov identifier NCT02299713 (11 Nov. 2014).

## Background

Osteoarthritis (OA) is the most common form of arthritis [1, 2]. Its prevalence is greater among women than men [3, 4]. Several factors contribute to the risk of OA, including age, gender, genetics, behavioral influences, obesity, injury, and reduced muscular strength [5]. Significant consequences of OA are activity limitations, reduced participation in work and social activities, and mental distress.

The most recent clinical guidelines based upon evidence from the UK National Institute of Clinical Excellence [6] and the Osteoarthritis Research International (OARSI) suggest that the treatment for OA be multidisciplinary in nature and consider non-pharmacological treatment (such as education, aerobic and resistance exercises, and weight loss) as well as pharmacological treatment options (paracetamol) when an additional treatment is required. In an OARSI systematic review, five of the eight guidelines considered acupuncture to be one of the 12 possible non-pharmacological therapy modalities for OA [7].

Acupuncture has been demonstrated to be a safe treatment with a low risk of serious side effects [8–12]. OA guidelines [13, 14] emphasize treatment safety as an important issue, especially when the therapeutic agents for elderly patients with knee OA and comorbidities are concurrent drugs that could have serious, life-threatening interactions.

Acupuncture could be used to explain the roles that the hypothalamic-pituitary-adrenal (HPA) axis and the endogenous opioid (EO) system play as important mediators of pain, the stress response, and other stimuli [15]. Recent neuroimaging studies by positron emission tomography have shown that the relationship between the neurochemical imbalance in the EO system [16] and the long-term pain relief produced by acupuncture practice has the opposite results of those observed with placebo (sham) acupuncture and that only acupuncture exerts a long-term effect through the EO system [17].

The effect of acupuncture on the HPA system is far from clear. Although cortisol release is one of the consequences of HPA system activation, different studies have shown opposite responses of cortisol release in response to acupuncture. One mediating factor may be the presence or absence of anxiety and depression, which are known to predict the onset and severity of chronic pain [18].

Although many studies have demonstrated the efficacy of acupuncture in the treatment of OA-associated knee pain [19], only one study [20] demonstrates the effectiveness of electro-acupuncture (EA) for pain reduction that includes the indicative reduced cortisol levels and elevated blood beta-endorphins within the same patient group. It would be of great interest to determine whether the lack of these kinds of results could be masked by interfering factors such as anxiety and depression.

When acupuncture was compared with some control groups (waiting list and control groups with active treatment), the positive effects reached the threshold of clinical relevance. Moreover, the only non-pharmacological therapeutic treatment for OA shown to reduce pain and improve physical function was exercise, and this treatment either closely met or exceeded the threshold for clinical relevance [21].

Although many studies show the efficacy of acupuncture for OA pain, the lack of a common methodology for the trial design complicates the possibility of comparing the respective results in order to determine treatment efficacy.

In this work, we have collected the following methodological suggestions for acupuncture studies that would minimize the design biases: the number of patients recruited to achieve the significant differences, treatment number, long-term treatment [22, 23], recruitment blinding method, EA use [24, 25], assessment of patient perceptions about the type of technique used before and after treatment [26], the objective and subjective assessment of the technique, and finally the use of sham with no needle penetration (a non-penetrating sham control). Thus, our aim is to apply these methods to verify the effectiveness of acupuncture for OA pain. Furthermore, our placebo use would permit the validation of the hypothesis.

Additionally, the current economic reality that dictates the need to develop favorable cost-benefit therapies for both patients and the health-care system should not be overlooked, and acupuncture is a cost-effective therapy for the treatment of knee OA [27].

Finally, a great deal of evidence indicates that various possible physiological mechanisms explain how acupuncture may provide pain relief, and these indicate that further research is needed to clarify the interrelationship and regulation of these mechanisms.

Although cortisol release is one of the consequences of HPA system activation, some studies have shown responses opposite to the cortisol release produced by acupuncture practice. Normal individuals show opposite cortisol responses to those with chronic pain conditions. This suggests that if the HPA axis contributes to the efficacy of acupuncture, its role is more complex than simply deactivating this system. Rather the contribution may depend on the population being studied. One of the objectives of this study is to provide more data to elucidate possible mechanisms of action and to show correlations between reductions in pain and changes in hormonal markers of HPA axis function. This will aid in establishing whether changes in HPA axis are causal or merely epiphenomenal in treatment efficacy.

This following study is a project to assess the effectiveness of EA for the treatment of common pathologies that involve chronic OA pain in knee. Additionally, it will evaluate pain perception, plasma endorphin and cortisol levels, patient quality of life, and the need for pharmacological drugs regardless of whether EA is applied.

### Hypothesis

#### Conceptual hypothesis

Patients who receive EA will have, to some degree, better pain control than non-treated patients.

#### Operative hypothesis

Patients who receive EA will have a measured basal pain perception that is three points less on the visual analogue scale (VAS) after EA treatment in comparison with non-treated patients.

#### Statistical hypothesis

Practice of EA will result in a statistically significant control of pain compared with the pain levels of non-treated patients.

## Methods/design

### Trial design

**Fig. 1:**
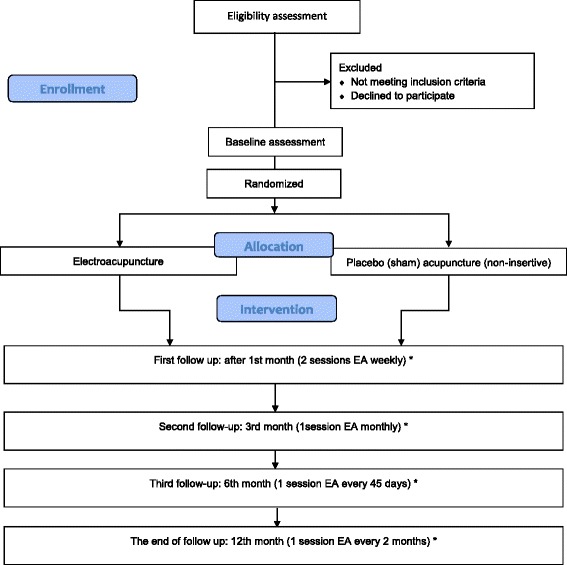
Trial flow. The study trial flow is described, indicating the patient selection process, treatment, and follow-up. *Visual analogue scale, Western Ontario and McMaster Universities Osteoarthritis Index, Short Form 36 version 2 health survey, blood sample collection, Goldberg Anxiety and Depression Scale, and medication use are measured on each follow-up visit. EA, Electro-acupuncture

This study proposes a randomized, double-blind, placebo-controlled, pre- and post-test, parallel design clinical trial which conforms to the CONSORT (Consolidated Standards of Reporting Trials) [28] and STRICTA (Standards for Reporting Interventions in Clinical Trials of Acupuncture) guidelines [29] for acupuncture studies (Fig. 1).

### Participants

Participants will be selected from a population with knee chronic pain caused by OA (>3 months in duration), from the communities of the island of Mallorca (Balearic Islands, Spain). These participants will be recruited in the outpatient clinics of the island’s hospitals and advertisements in local newspapers and through the hospital website (http://www.hsll.es). Advertisements for patient recruitment will not specify acupuncture as one of the treatments being investigated or offered. Doctors from the trauma services of local hospitals will be invited to screen patients. The clinical trial will be performed by the Pain Unit at Son Llàtzer Hospital, Palma de Mallorca.

A total of 160 patients will be necessary (80 subjects for each treatment group) to detect differences of at least three points in the pain perception assessment according to VAS (scale of 0 to 10). Accepted values will be for an alpha risk of 1 % and beta risk of less than 5 % in a bilateral contrast as well as a value of 4 for the standard deviation (size effect of 0.75). It is assumed that 20 % of the trial patients will be lost to attrition. Patients will be included in the study by case-consecutive, non-probability sampling after responding to a recruitment visit to the Pain Clinic; then if they sign an informed consent form, they will be placed randomly into one of the treatment groups [30].

### Inclusion criteria

Eligibility requirements will include the following: male and female patients who are more than 50 years old and who have knee OA diagnosed according to the American College of Rheumatology classification criteria (with at least one osteophyte in the tibiofemoral joint and with a Kellgren-Lawrence grade of more than 2), who have had a history of moderate or greater knee pain of more than 3 months’ duration and have reported moderate or greater, clinically significant, knee pain during most days of the last month before the interview, and who have given their consent to participate in the study. Patients will be selected among those who are not on the waiting list for total knee arthroplasty by the traumatology unit of our hospital.

### Exclusion criteria

Patients with any of the following will be excluded from the study: a history of any secondary OA associated with any systemic arthropathy (i.e., rheumatoid arthritis or gouty arthritis); any knee treatment with steroids, methotrexate, or azathioprine; recent traumatisms caused by acupunctural insertion; a history of bleeding disorders or any disease related to acupuncture contraindications; a history of any knee injection in the previous 6 months (e.g., cortisone and hyaluronic acid); an acupuncture treatment history; or a history of morphine or morphine derivative use.

### Removal criteria

Patients will be removed by voluntary removal or by failure to consecutively attend two or more EA sessions.

### Variables

#### Main variables

The primary outcomes will be the changes from the baseline of the VAS and the Western Ontario and McMaster Universities Osteoarthritis Index (WOMAC) for pain at the completion of treatment at 12 weeks.

#### Secondary variables

Secondary variables to be considered are the following: the change in the secondary efficacy variables from the baseline of the Short Form 36 version 2 health survey (SF-36v2) (patient-perceived quality of life), the change in plasma cortisol and beta-endorphin levels, the scores for the Goldberg Anxiety and Depression Scale (GADS), changes in pain medication use, changes in functional capacity and stiffness (WOMAC subscales), and VAS scores measured at 1 month, 3 months, 6 months, and 1 year after study commencement.

### Procedure

Figure 1 describes the trial protocol. The eligibility of prospective participants will be determined by a researcher who is not involved in the assessment or treatment of the participants. Ethical approval was obtained from the Balearic Islands Human Research Ethics Committee in July 2013 (CEI-IB, #IB 2077/13 PI).

Upon arrival at the Clinic for Pain Treatment, recruited patients will be informed once again about the trial objectives, and any questions or doubts that the patient might still have with respect to the study will be resolved. Patients will then be informed that they are testing a new EA technique for knee pain treatment. Placebo or sham acupuncture (SA) will not be mentioned to the patient during the interview or referred to in the informed consent form. Subjects will be placed into one of two study groups (with a strict 50 % probability), and to reinforce and encourage treatment follow-up, patients will be told that the effects are to be perceived over the medium- to long-term time frame. Patients will be re-assessed to ensure that they still comply with the selection criteria and that they have not acquired any new criteria for exclusion. Participants will provide informed consent in the first baseline visit (Table 1).

**Table 1:** Planned visit schedule for treatment and follow-up, indicating visit number and frequency

	Visits									
	0	1st	2nd -7th	8th	9th	10th	11th	12th	13th-14th	15th
			(2 weekly /month)	(follow-up 1st month)	(2nd month)	(follow-up 3rd month)	(every 45 days)	(follow-up 6th month)	(every 2 months)	End of follow-up (1 year)
**Patients**										
Patient’s evaluation and collection of the relevant data	√									
Inclusion/Exclusion criteria	√									
Explanation of the objectives of the procedure and how it works	√									
Informed consent	√									
Randomization and blinding		√								
Expectation of acupuncture				√		√		√		√
**Treatment**										
Electro-acupuncture (EA)		√	√	√	√	√	√	√	√	√
Sham acupuncture (SA)		√	√	√	√	√	√	√	√	√
**Outcomes**										
Visual analogue scale (VAS)	√			√		√		√		√
McMaster Universities Osteoarthritis Index (WOMAC)	√			√		√		√		√
Medication use	√			√		√		√		√
Goldberg Anxiety and Depression Scale	√			√		√		√		√
Short Form Health Survey (SF-36v2)	√			√		√		√		√
Blood sample collection	√			√		√		√		√
**Trial evaluation**										
Adverse event				√		√		√		√
Outcome assessment										√
Reasons of drop-outs or withdrawals				√		√		√		√
Satisfaction and expectations Survey										√

### Baseline visit

The researcher will re-collect clinical data of patients, including age, sex, and body mass index. In the baseline interview, the participants will not yet be assigned to a treatment group.

- *Pain perception assessment* will be based on (1) the VAS score and (2) the WOMAC scale pain score. The VAS is a continuous scale made up of a 10-cm, unmarked, horizontal line, upon which the subject makes a hash mark depending on the amount of pain felt; a mark on the far left of the line indicates “no pain” (score of 0), and a mark to the far right qualifies as “pain as bad as it could be” or “unbearable pain” (score of 100). The WOMAC scale is used to evaluate the condition of patients with OA of the knee and the hip and includes pain, stiffness, and physical functioning of the joints. This scale measures five items for pain (with a score range of 0–20), two for stiffness (score range of 0–8), and 17 for functional limitation (score range of 0–68). The physical functioning questions cover everyday activities. These scales will be used separately and will not be summed. Patients will respond orally to the five levels with the following criteria: “none” = 0, “a bit” = 1, “quite a bit” = 2, “a lot” = 3, and “very much” = 4. If two or more questions are left unanswered, the scale will be declared invalid. If the patient does not respond to one question, a mean will be taken from the results of the other questions. The range will be 0–98.
- *Analgesic medicine use*. This questionnaire will have four questions developed according the EUROHIS (European Health Interview Survey) recommendations in order to harmonize the information regarding medicine use [31]. Subjects will be asked (1) about the prescription medicine their general practitioner may have prescribed for them (“Have you taken any pain medicine prescribed by your general practitioner?”) as well as any medication not prescribed by their general practitioner (“Have you taken any pain medicine not prescribed by your general practitioner”) and (2) whether or not their prescribed and non-prescribed pain medication use has increased or decreased.
- *The degree of functionality and stiffness* according to the WOMAC scale. There are two questions for stiffness (scale of 0–8) and 17 questions for functional limitation (score range of 0–68). Each question will be answered orally with the following criteria: “none” = 0, “a bit” = 1, “quite a bit” = 2, “a lot” = 3, and “very much” = 4. If two or more questions are unanswered, the scale scores will be declared invalid and not counted.
- *Anxiety/depression level* according to the GADS. This consists of two subscales (of nine questions for each scale): an anxiety scale (1–9) and a depression scale (10–18). The first questions for each scale (1–4 and 10–13) are preconditioned questions to determine whether the patient should attempt to answer the rest of the questions of the two subscales. Effectively, at least two questions must be answered affirmatively in the first scale (1–4) for the patient to continue; in the second scale, one answer is sufficient for questions 10–13 for the patient to continue with the rest of the questions. This study will use the Spanish version validated by Montón et al. [32].
- *Degree of quality of life*. This will be assessed according to the SF-36v2 questionnaire and is compounded by 36 items, which yield an 8-scale profile of functional health and well-being scores as well as psychometrically based physical and mental health summary measures and a preference-based health utility index. The Spanish version of the SF-36 questionnaire by Ware and Sherbourne [33] has been correctly translated and standardized and has been given the Spanish title of *Cuestionario de Salud* SF-36 [34].

The first baseline plasmatic sample will be extracted from the antecubital vein to evaluate beta-endorphin and cortisol levels, and the sampling time will be between 9 and 14 h. To avoid any diurnal variation in plasmatic hormonal levels, subjects will always be assigned the same hour for the extractions. Five blood samples (5 ml each) from each patient will be taken under aseptic conditions from the antecubital vein; one extraction will required before the treatment, one at 1 month after the start of treatment, one at 3 months, one at 6 months, and one at the end of treatment. Except for the first pre-treatment sample, extractions will take place 5 min after finishing the acupuncture treatment. Once extracted, blood will be placed in a refrigerated tube with EDTA (ethylenediaminetetraacetic acid) anticoagulant and centrifuged immediately. Plasma will be extracted with a micropipette and divided in two equal fractions (one for the cortisol and one for the beta-endorphin analysis). Samples will be stored at −70 °C until the analysis. The baseline interview treatment will have no assigned sampling time and will occur on the first day, when the subject is assigned to his or her respective treatment group.

### First day of treatment

#### Randomization

Each eligible patient will be randomly assigned to one of two groups: “A” and “B”. A random number list generated by SPSS statistical software version 18.0 (IBM Corporation, Armonk, NY, USA) (balanced for each six cases per study branch) will be used for the allocation to each group. Researchers from the statistical center will send the randomized list in a numbered, sealed, and opaque envelope to the researcher responsible for participant recruitment and group assignment.

#### Blinding

The patient codes of the double-blind study will be placed in numbered, sealed, and opaque envelopes. Researchers, personnel performing the interviews, statisticians, and participants will be blinded to patient allocation. Study groups will consist of the following, although patients will have no knowledge into which study branch they have been placed: the experimental group (A) receiving EA or the control group (B) receiving SA.

#### Intervention

The treatment will be applied in eight sessions (two per week) for 1 month. Group A patients will receive the EA treatment, and group B will receive the SA treatment. Both groups will receive two weekly sessions for 4 weeks, then one session (each month) for the first 3 months, one session (each 45 days) until 6 months, and finally one session every 2 months until finalizing the study after a year’s time.

Treatments will be performed by the same acupuncturist, an anesthesiology specialist who has studied Traditional Chinese Medicine (TCM) at the University of Chinese Medicine, Beijing, China, holds a master’s degree in acupuncture from the University of León, Spain, and has more than 8 years of clinical experience in therapeutic pain management by acupuncture.

##### Experimental group (group A)

EA will be performed for a 20–30 min treatment session, and the patients will be semi-seated comfortably on a stretcher. A 0.30 × 30 mm needle will be used and will make a 10–30 mm insertion into the skin, depending on the patient’s constitution. Needle insertion points will be selected according to the TCM meridian theory for knee pain treatment. Upon insertion, EA will be applied by a biphasic pulse generator apparatus at a frequency of 3 Hz and at the maximum tolerable intensity. Table 2 shows the local and distal points: St 34 (*Liáng Qiū*), localized 2 cun proximal to the upper lateral border of the patella, in a groove of the vastus lateralis muscle; St 35 (*Dú Bí*), in the depression inferior to the patella and lateral to the patellar ligament; St 36 (*Zú Sān Lĭ*), one fingerbreadth lateral to the anterior crest of the tibia, on the tibialis anterior muscle; Lv 8 (*Qū Quán*), proximal to the medial end of the popliteal crease, in a depression anterior to the tendons of the semitendinosus and semimembranosus muscles; Sp 9 (*Yīn Líng Quán*), in a depression distal to the medial condyle of the tibia, at the junction of the shaft and the medial condyle; Sp 10 (*Xuè Hăi*), 2 cun proximal and slightly medial to the medial superior border of the patella, in a depression on the vastus medialis muscle; Ki 10 (*Yīn Gŭ*), at the medial end of the popliteal crease, between the tendons of the semimembranosus and semitendinosus muscles, on the level of the knee joint space; M-Le 16 (*Xi Yan*), inferior to the patella, medial and lateral to the patellar ligament; and M-Le 27 (*Hè Ding*), in the center of the upper border of the patella. As a distal point, St 44 (*Nèi Tíng*), between the second and third toes, proximal to the interdigital fold, will be used.

**Table 2:** Framework of acupuncture points that should be selected when administering acupuncture treatments

Location	Acupuncture points	Description
Local points		
	St 34 (*Liáng Qiū*)	2 cun proximal to the upper lateral border of the patella, in a groove of the vastus lateralis muscle.
	St 35 (*Dú Bí*)	In the depression inferior to the patella and lateral to the patellar ligament.
	St 36 (*Zú Sān Lĭ*)	One fingerbreadth lateral to the anterior crest of the tibia, on the tibialis anterior muscle.
	Lv 8 (*Qū Quán*)	Proximal to the medial end of the popliteal crease, in a depression anterior to the tendons of the semitendinosus and semimembranosus muscles.
	Sp 9 (*Yīn Líng Quán*)	In a depression distal to the medial condyle of the tibia, at the junction of the shaft and the medial condyle.
	Sp 10 (*Xuè Hăi*)	2 cun proximal and slightly medial to the medial superior border of the patella, in a depression on the vastus medialis muscle.
	Ki 10 (*Yīn Gŭ*)	At the medial end of the popliteal crease, between the tendons of the semimembranosus and semitendinosus muscles, on the level of the knee joint space.
	M-Le 16 (*Xi Yan*)	Inferior to the patella, medial and lateral to the patellar ligament.
	M-Le 27 (*Hè Ding*)	In the center of the upper border of the patella.
Distal point		
	St 44 (*Nèi Tíng*)	Between the second and third toes, proximal to the interdigital fold.

The needles will be inserted through the same guide tube that will be used for the control group. The objective is for control patients to perceive the De-Qi sensation, which is like a tingling or numbness on the specific points punctured. Subsequently, EA will be used between *Xiyan* points and also to the points that the patient recalls as the area of pain. If both knees are affected, the same acupuncture points will be applied.

To ensure that the treatment procedures for the treatment and the control groups are as similar as possible, we will tape two guide tubes to two sham points at the abdominal area, approximately 3 cm lateral to and slightly above the umbilicus bilaterally. Immediately after, we will affix a pair of needles by adhesive tape to the surface at these same points though without needle insertion. This site was chosen for two reasons. First, these sites were at two meridians that are theoretically irrelevant to knee pain. Second, to facilitate blinding, we wanted to provide the participants of both groups the opportunity to be able to feel a sensation similar to the actual insertion of the needle.

##### Control group (group B)

A valid method of placebo acupuncture with non-insertion of the needle will be used for the control group [35]. Two needles will be inserted into the sham points of the abdominal area in the same position used for group A, approximately 3 cm lateral to and slightly above the umbilicus bilaterally, and then two pieces of adhesive tape will be immediately taped next to the needles. In addition, a mock plastic needle guide tube will be placed on the surface of each of the nine true points in the leg so that some discernable sensation will be produced; then a needle will be immediately applied without needle insertion to the dermal surface at each point and will be retained at this position for 20–30 min. These plastic guide tubes will be applied in the same manner as for group A in order to produce the same perceptible sensation.

The control group will receive the same session and schedule as group A. This will include the same active needle placements, although needle insertion will not occur at these nine points (local and distal points). Not only will the accessories of the EA equipment be connected in the same point in group A without electric stimulation, a mock transelectrical stimulation unit (which emits a “timer” sound and has a blinking light) will be attached to the sham needles at the knee.

To facilitate patient blinding for both groups, a screen will be placed below the abdomen to prevent participants from actually observing true or sham procedures at the knee area but at the same time will permit them to see the procedure that is being given in the abdominal area. It is important to state that this type of intervention will need some time for the results to be noted, and a thorough follow-up is necessary to avoid dropouts from both groups.

### Next several visits for treatment (two sessions per week until the completion of eight sessions)

The same treatment will be repeated according to group. In the last session, blood samples will be drawn 5 min after the treatment completion, at the same time of day as the previous extraction.

### At 1 month’s time

The interviewers will repeat the assessments for pain level, analgesic consumption, and the type of treatment that the patient believes he or she is receiving as well as the assessment by the WOMAC scale and the GADS. The patient will once again answer the quality-of-life questionnaire (SF-36v2). Once the forms have been completed, treatment will be administered to the patient.

### Subsequent visits

At 3 and 6 months after treatment start and at the final visit, the questionnaires again will be filled out. The interviewers will also note EA-related adverse effects or complications observed either by the participants or by the acupuncturist throughout the duration of the treatment. Treatments will be performed each month up until the third month, and after this latter treatment session, with just one treatment every 45 days until the sixth month. Finally, a treatment will occur every 2 months until the study is completed at the end of 1 year. Blood tests will be performed again on the third month, on the sixth month, and at the end of treatment period.

### Study end

At study completion, a questionnaire will be given that includes questions related to patient satisfaction with the treatment received, the type of treatment patients believe they have received, and their expectations for improvement.

### Statistical analysis

The results will be analyzed by using the SPSS statistical software version 18.0. A descriptive analysis of the collected data will be obtained by using the mean, the standard deviation, and the maximum and minimum values for the quantitative variables that follow a normal distribution. For variables without a normal distribution (degree of pain, plasma levels, and quality of life, among others), the median, the 25th and 75th percentiles, and maximum and minimum values will be calculated.

Qualitative variables will be expressed as frequencies and percentages. Differences shall be detected by chi-square or Fisher tests for categorical variables and Student *t* or Mann-Whitney tests for the resultant variables.

## Discussion

The aim of the study is to measure the effectiveness of EA in alleviating OA knee pain. Effectiveness indicators should be the relief of pain, stiffness and functional disability of the knee, the improvement of quality of live and a reduction in medication use.

The principal challenge expected with this study is the difficulty of effectively blinding SA application in a manner that is believable or credible for the patient. For this reason, we will avoid using the terms “sham acupuncture” or “placebo” in the information letter and consent form. The attempt to blind the process will be done by explaining to the patient that a new pain treatment will be assessed and compared with other techniques (infiltrations and analgesic stimulation) and that the selected patients must have no history of acupuncture treatment. The literature reports that the non-penetrating SA method has been widely used and has also been validated for avoiding skin receptor stimulation in SA [36]. Disclosure of a patient’s group affiliation will be avoided so that they will not be able to share information among themselves or compare treatments. The only person who will have full knowledge of the applied treatment will be the acupuncturist, although he will remain blind to the rest of the details of the study.

A satisfaction questionnaire will be completed at the end of the study to assess whether the patients believe they have or have not received treatment. To avoid introducing bias that could be caused by the interference of the circadian rhythm in the beta-endorphin and cortisol levels, patients will always be scheduled for blood sampling at the same hour.

As has been shown, it would seem clear that the treatment of knee OA must be a multidisciplinary approach, as there is currently no treatment alone that may be considered beneficial in absolute terms.

Although there are studies showing the efficacy of acupuncture for the treatment of OA pain, the lack of a common methodology to apply the treatment design complicates the comparison of the results between studies.

In this article, we combine all of the methodological suggestions for acupuncture studies, attempting to minimize the biases that may result from study design. We intend to make a comparison with a placebo that would allow the hypothesis to be validated.

We must not forget the current economic circumstances that drive us to look for therapies that are cost-beneficial for both the patient and the health system. In this direction, there are studies that suggest acupuncture as a cost-effective therapy for the treatment of knee OA.

Finally, sufficient evidence about the different physiological mechanisms would encourage further research on how acupuncture can relieve pain. However, the effect of acupuncture on the HPA system is not clear, and therefore we intend to provide more information that would clarify its function.

### Trial status

The trial is currently in the recruitment phase. Participant recruitment started in January 2015 and is expected to end in December 2015.

## Abbreviations

EA: Electro-acupuncture
EO: Endogenous opioid system
GADS: Goldberg Anxiety and Depression Scale
Group A: Experimental group
Group B: Control group
HPA: Hypothalamic-pituitary-adrenal
OA: Osteoarthritis
OARSI: Osteoarthritis Research International
SA: Sham acupuncture
SF-36v2: Short Form 36 version 2 health survey
TCM: Traditional Chinese Medicine
VAS: Visual analogue scale
WOMAC: Western Ontario and McMaster Universities Osteoarthritis Index
